# Probiotics and the Role of Dietary Substrates in Maintaining the Gut Health: Use of Live Microbes and Their Products for Anticancer Effects against Colorectal Cancer

**DOI:** 10.4014/jmb.2403.03056

**Published:** 2024-08-08

**Authors:** Yi Xu, Xiahui Wu, Yan Li, Xuejie Liu, Lijian Fang, Ziyu Jiang

**Affiliations:** 1 Phase I Clinical Cancer Trial Center, The Affiliated Lianyungang Hospital of Xuzhou Medical University, Lianyungang, 222002, P.R. China; 2 Department of Oncology, The Affiliated Lianyungang Hospital of Xuzhou Medical University, Lianyungang 222002, P.R. China; 3 Department of Oncology, The First People’s Hospital of Lianyungang, Lianyungang 222002, P.R. China

**Keywords:** Gut microbiome, eubiosis diet, probiotics, antitumor

## Abstract

The gut microbiome is an important and the largest endocrine organ linked to the microbes of the GI tract. The bacterial, viral and fungal communities are key regulators of the health and disease status in a host at hormonal, neurological, immunological, and metabolic levels. The useful microbes can compete with microbes exhibiting pathogenic behavior by maintaining resistance against their colonization, thereby maintaining eubiosis. As diagnostic tools, metagenomic, proteomic and genomic approaches can determine various microbial markers in clinic for early diagnosis of colorectal cancer (CRC). Probiotics are live non-pathogenic microorganisms such as lactic acid bacteria, *Bifidobacteria*, *Firmicutes* and *Saccharomyces* that can help maintain eubiosis when administered in appropriate amounts. In addition, the type of dietary intake contributes substantially to the composition of gut microbiome. The use of probiotics has been found to exert antitumor effects at preclinical levels and promote the antitumor effects of immunotherapeutic drugs at clinical levels. Also, modifying the composition of gut microbiota by Fecal Microbiota Transplantation (FMT), and using live lactic acid producing bacteria such as *Lactobacillus*, *Bifidobacteria* and their metabolites (termed postbiotics) can contribute to immunomodulation of the tumor microenvironment. This can lead to tumor-preventive effects at early stages and antitumor effects after diagnosis of CRC. To conclude, probiotics are presumably found to be safe to use in humans and are to be studied further to promote their appliance at clinical levels for management of CRC.

## Introduction

The microbes linked to the human gut are termed as gut microbiome and has existed and evolved over quite a few generations. With an enhanced interface of 250 to 400 m^2^, the gut is home to the existence of a lot of external factors and antigens. On-an-average, sixty tons of food passes through the gastrointestinal tract (GI tract) in a person’s life time. The microbial abundance of the gut is estimated to be more than 10^14^, which is 10-fold higher than the volume of human cells [1]. The gut microbiome is a vital and the largest endocrine organ associated with the microbes of the digestive tract and is a greatest modulator of the wellbeing and disease status of a human host [2, 3]. It is composed of a group of bacterial, viral and fungal communities along with their genetic material [4].

It is well established that the gut microbiome of healthy individuals and patients with colorectal cancer (CRC) vary significantly. It signifies the limited abundance of useful microbes or commensals and higher abundance of pathogenic or pro-carcinogenic organisms [5]. The uniqueness of CRC is its close association with gut microbiota. Therefore, gut dysbiosis is a hallmark of neoplastic transformation of colorectal cells with decrease in occurrence of diverse organisms and onset of the gut enriched with tumorigenic organisms [6]. Consequently, the manner in which CRC progresses is dependent on the composition of the gut microbiome [7]. Therefore, the humans have been engaged in a symbiotic relationship with a group of gut microbiome and this group can be influential in beneficial or detrimental effects on the host health [8].

Probiotics are live non-pathogenic microorganisms that can help alleviate the dysbiosis-associated symptoms in an affected gut and provide other beneficial effects in an individual when administered in sufficient volumes [9, 10]. Probiotics meaning ‘of life’ in Greek can pose health benefits on intestinal microbial flora, improve bowel stability and minimize the detrimental effects on the host [11]. Bacteria such as lactic acid bacteria, *Bifidobacteria*, *Firmicutes* and yeast are potentially used as probiotics. In this regard, the *Firmicutes*/Bacteroidetes ratio is critical for maintenance of gut homeostasis as an altered ratio could lead to dysbiosis [12, 13]. Serving this purpose, fermented products like yogurt and other dietary supplements like milk, chocolate products and condensed milk are effective in being used as a probiotics as they are rich in useful microbes. In such cases, fermented foods become delivery vehicles for these beneficial bacteria [14-16].

Among the commonly studied probiotics, lactic acid bacteria such as *Carnobacterium*, *Enterococci*, *Lactobacilli*, *Lactococci*, *Leuconostoc*, *Oenococcus*, *Pediococci*, *Streptococci*, *Tetragenococci*, *Vagococcus* and *Weissella* are consumed as a part of human diet. Other bacterial species like *Bifidobacteria*, *Bacillus* and *Escherichia*, including the fungal species *Saccharomyces* are also widely used as probiotics [17, 18]. Among the yeasts and molds those are consumed as probiotics, *S. cerevisiae* and *S. boulardii* are prominent ones [19]. Therefore, the current review focuses on enlisting the microbial markers for CRC, the effects of dietary intake on gut microbes along with the use of probiotics and their metabolites for cancer therapy.

## A list of Microbial Markers for CRC as Determined by Multiomics

### Metagenomic Markers

Among the microbial markers for CRC, *Actinomyces*, *Bacteroides fragilis*, *Bifidobacterium*, *Clostridium hylemonae*, *C. symbiosum*, *Enterococcus faecalis*, *Escherichia coli*, *Fusobacterium nucleatum*, *Gemella morbillorum*, *Lachnoclostridium*, *Parvimonas micra*, *Peptostreptococcus stomatis*, *Porphyromonas asaccharolytica*, *Pseudomonas*, *Roseburia*, *Ruminococcus*, *Salmonella*, *Solobacterium moorei* and *Streptococcus bovis* have been found to be enriched in the gut and feces of patients with CRC. *Treponema denticola* and *Prevotella intermedia* are oral microbiota associated with CRC, whereas, serum antibodies were identified against *Streptococcus gallolyticus* [20-22].

Significantly, some organisms were identified to exist predominantly in fecal samples of CRC patients in comparison to the patients with adenomas. These organisms belong to Phylum *Firmicutes*, (Class *Clostridia*: Family *Lachnospiraceae*, *Ruminococcaceae*, *Erysipelotrichaceae*, *Peptostreptococcaceae*, *Christensenellaceae*, *Defluviitaleaceae*, *Clostridiaceae*; Class *Bacilli*: Family *Streptococcaceae*; Class *Negativicutes*: Family *Veillonellaceae*; Class *Erysipelotrichia*: Family *Erysipelotrichaceae*), Phylum *Bacteroidetes* (Class *Bacteroidia*: Family *Bacteroidaceae*, *Rikenellaceae*, *Porphyromonadaceae*), Phylum *Proteobacteria* (Class *Pasteurellales*: Family *Pasteurellaceae*; Class *Gammaproteobacteria*: Family *Enterobacteriaceae*), Phylum *Synergistetes* (Class *Synergistia*: Family *Synergistaceae*), Phylum *Actinobacteria* (Class *Actinomycetia*: Family *Bifidobacteriaceae*) and Phylum *Fusobacteria* (Class *Fusobacteriia*: Family *Fusobacteriaceae*) [23].

It was evident from another sequencing analysis that microbes of genera *Bacteroides*, *Roseburia*, *Ruminococcus*, *Oscillibacter*, *Alistipes*, *Akkermansia*, *Halomonas*, *Shewanella*, *Faecalibacterium*, *Blautia* and *Clostridium* were abundantly observed in the CRC patients. Also, *Firmicutes*, *Bacteroidetes*, *Proteobacteria*, *Fusobacteria* and *Actinobacteria* were observed in saliva, feces and cancer tissues of CRC patients. Also, a list of microbial populations was dominant in one continent in comparison to another continent. Yet, the flora of genera *Parvimonas*, *Peptostreptococcus*, *Fusobacterium*, *Alistipes*, and *Escherichia* were commonly observed in population across most continents studied. Also, the members of *Streptococcus* species, *Helicobacter pylori*, *Enterococcus faecalis*, *Bacteroides fragilis*, *Clostridium septicum* and *Escherichia coli* were identified in cancer tissues of CRC patients [24] (Table 1).

### Proteomic Markers

Along with the identification of microbiome specific for CRC, pre-diagnostic protein markers such as serum carcinoembryonic antigen (CEA), basigin (CD147) and glycoprotein A33 (GPA33) have also been identified in accordance with the occurrence of CRC [25, 26], besides Actin Beta Like 2 (ACTBL2), Dipeptidase 1 (DPEP1), fibroblast growth factor 21 (FGF-21) and pancreatic prohormone (PPY) [27-29]. In addition, BAG cochaperone 4 (BAG4), Interleukin 6 receptor (IL6R), Von Willebrand factor (VWF) and epidermal growth factor receptor (EGFR) proteins have also been associated with the CRC diagnosis [30]. Also, clusterin, proteasome subunit alpha type 1 (PSA1), leucine aminopeptidase 3 (LAP3), annexin A3 (ANXA3), maspin (serpin B5), olfactomedin 4 (OLFM4), CD11b, integrin α2 (ITGA2), periostin, thrombospondin-2, serine/threonine kinase 4 (STK4), S100 calcium-binding protein A9 (S100A9) and macrophage mannose receptor 1 (MRC1) were overexpressed in primary CRC tumors [31-35].

Among prognostic markers for CRC, human leukocyte antigen B (HLA-B), 14-3-3 phospho-serine/phospho-threonine binding proteins, A disintegrin and metalloproteinase with thrombospondin motifs 2 (ADAM-TS2), latent transforming growth factor beta binding protein 3 (LTBP3), Nucleoside diphosphate kinase B (NME2), Jagged Canonical Notch Ligand 2 (JAG2), collagen type XII protein and collagen-derived urine AGP peptide are significant ones [36-38]. Also, collagen VI, inositol polyphosphate-4-phosphatase, and Maspin are to be critically mentioned for use as prognostic factors linked to CRC recurrence [39]. Although these proteins will be further studied for clinical use, CEA remains the widely used protein at the clinical level for diagnosis of CRC to date.

### Genomic Markers

As genomic markers, mutations of tumor suppressors such as Adenomatous polyposis coli (APC), Tumor protein P53 (TP53), Mothers against decapentaplegic homolog 2 (SMAD2/4), Netrin receptor DCC besides the mutations of proto-oncogenes such as Kirsten rat sarcoma virus (KRAS), Catenin beta-1 (CTNNB1), v-raf murine sarcoma viral oncogene homolog B1 (BRAF) and phosphatidylinositol-4,5-bisphosphate 3-kinase catalytic subunit alpha (PIK3CA) along with the dysregulation of Wnt, TGFβ/BMP, RTK/Ras, PI3K/Akt pathways are widely regarded as markers for CRC onset [40, 41]. Among the methylation markers, SEPTIN9, EHD3, TMEM240, SMAD3, and NTRK3 are the crucial ones [42].

## The Effects of Intake of Dietary Fibers on Gut Microbiome

Humans who consume high-fiber diet are less prone to chronic disorders including cancer [43]. Although the benefits of taking a diet rich in fiber has been linked to intense health benefits, the recommended daily levels of 20 to 35 g is not reached per day as a nominal 15 to 26 gms is consumed in most countries. As end-products of fermentation of fibers by specific bacteria, lactate, succinate, gases including hydrogen and SCFAs (acetate, propionate and butyrate) are released at large. Hence, the intake of prebiotics can enhance the abundance of specific probiotic bacteria responsible for production of SCFAs. These organisms may include *Bifidobacterium*, *Faecalibacterium*, *Ruminococcus*, *Lactobacillus*, *Akkermania* and *Roseburia*. Therefore, a fiber-rich diet can improve the overall health of an individual and the initial abundance of a particular microbiota can produce prolonged beneficial effects [44].

In this regard, 40 to 45% of everyday caloric intake of humans is linked to carbohydrates, with plant-based carbohydrates contributing to 50 to 60% of the intake. It is assumed that an average 30 gms of carbohydrates reach the colon. The daily protein intake ranges between 70 to 100 gms [45, 46]. Also, everyday a diet rich in red meat (23 gms), processed meat (2 gms), sugar-sweetened beverages (3 gms), sodium (3 gms) and trans fatty acids (0.5%) is consumed across countries, whereas, diets with nutrients lower than optimal are consumed (fiber- 24 gms; calcium- 1.25 gms; omega-3 fatty acids- 250 mg; polyunsaturated fatty acids- 11% of daily energy requirement) as a daily routine [47]. It is also important to note that diet rich in fruits and vegetables can provide a fiber content of 60 g/day [48].

It is also remarkable to note that gut microbiota have the capacity to become twice their volume over a period of 1 h and can vary every day based on the dietary intake. At the most, the microbial composition can vary to the family level significantly within 1 or 2 days. In a human circadian rhythm, 10% of Operational Taxonomic Units (OTUs) can fluctuate based on the type of diet. The epithelial histone deacetylase 3 (HDAC3) can alter lipid uptake of the intestine as a result of fluctuations induced by histone acetylation mediated by microbiota and induce obesity in affected humans. In addition, sleep disturbances and the duration of dietary intake contribute considerably to the composition of gut microbiome. Delayed intake of meal can disturb the microbial flora of saliva and result in pro-inflammatory activities. However, fiber intake alters the beneficial microbes to almost 15%in 24 h and can increase the volume of beneficial microbiota of the *Bifidobacterium* and *Lactobacillus* species [49].

## Anticancer Activity of Probiotics and Their Metabolites

The anti-tumorigenic activity of probiotics is based on mechanisms such as by modifying the composition of gut microbiota, changing the metabolic activities of probiotics, production of anticancer compounds such as conjugated linoleic acid, SCFAs and lactic acid, inhibiting the proliferation of cancer cells and inducing apoptosis in such cells. Also, the inhibition of carcinogenic factors and degradation of carcinogenic compounds, immunomodulation in cancer environment by in situ vaccination using probiotic constituents and improving the gut barrier can lead to anticancer effects of probiotics [50]. In patients suffering from CRC, supplementation with probiotics can exhibit anticancer effects by producing anticancer compounds for instance, butyrate. These live bacteria including *Bifidobacterium* and *Lactobacillus* spp., are known to exert such effects in the host [51, 52]. These bacteria can ferment fibers and proteins and result in production of SCFAs, in addition to generating vitamin K and a complex of B vitamins B_1_, B_7_ and B_9_ and other anti-inflammatory compounds [53].

### Modifying the Composition of Gut Microbiota for Enhanced Antitumor Immunity by Oral Intake and Fecal Microbiota Transplantation

The oral intake of useful bacteria in the form of probiotics through sources including dairy products such as yogurt, cultured buttermilk, and cheese besides non-dairy fermented substrates such as soy based products, cereals, legumes along with fish, breast milk and guts of animal species can decrease the abundance of pathogenic bacteria [54]. Suggestively, probiotics such as *Lachnospiraceae* species, *Bifidobacterium animalis* and *Streptococcus thermophiles* can prevent CRC from progressing to aggressive forms. This can prevent the colonization of pathogenic bacteria in the intestine and put a check on gut infections. The probiotic intake can also aid in conferring colonization resistance by virtue of competition for nutrients and reduces intestinal inflammation which can lessen the effects of CRC in preexisting patients. Yet, a matter of concern is horizontal gene transfer from probiotic bacteria resulting in antibiotic resistance among both useful and pathogenic bacteria [55].

The recolonization of the probiotic bacteria in the intestine can improve the TH1 helper cell response and enhance the effectiveness of immunotherapy. Also, mice that had their colons enriched with probiotic bacteria had reduced incidence of tumor and decreased tumor growth along with improved immune surveillance mediated by cytotoxic T lymphocytes. The antitumor T cell responses are found to be positively correlated to the presence of *Bifidobacterium*. The selective enhancement of these lactic acid bacteria in the gut can therefore enhance the incidence of antitumor T cell helper responses and decrease tumor growth by improving the resultant immune response. These microbes can survive the harsh and hypoxic TME due to the improved nutrient availability in regions that exhibit necrosis. Also, they can increase the intensity of mature dendritic cells, which can mimic antigen presenting cells and activate the functioning of T-cells. Also, immunotherapy using checkpoint inhibitors showed the abundant presence of bacterial genera that can prolong the progression-free survival [56]. In addition, causing an increase in SCFAs by the supplementation with probiotics can lead to decreased invasion of the intestine by pathogenic bacteria (reduces the infection percentage of *H. pylori* by around 13%) [57].

**Fig. 1:**
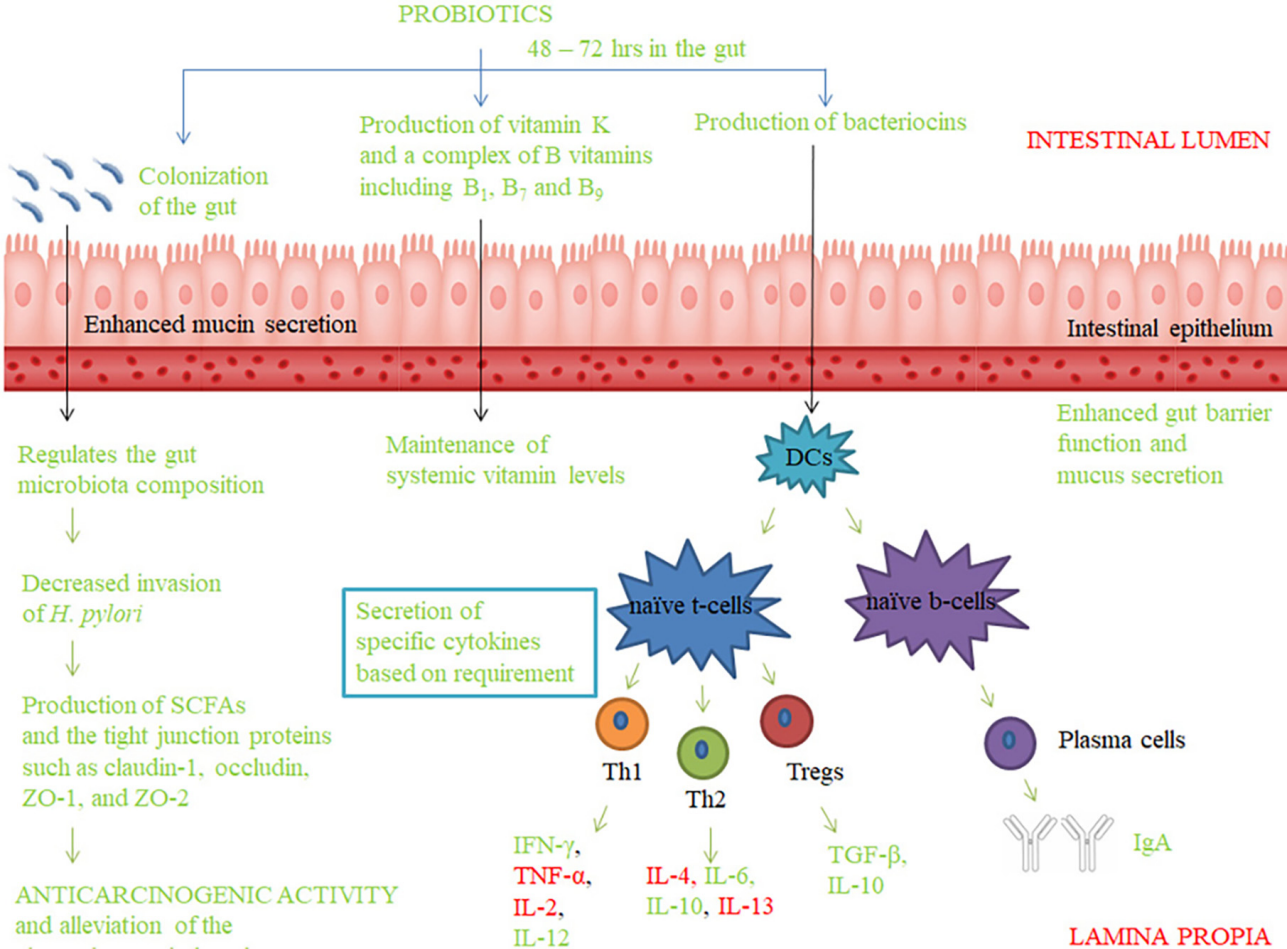
Probiotic supplementation and immunological effects in the gut.

Probiotics generally inhabit the gut and aid in enhanced expressions of mucin and secretion of the mucus, the layer in which the pathogen is neutralized by IgA. Later, the luminal constituents of the gut are taken up directly by the dendritic cells and macrophages that express TLR-6 and TLR-2, or via pinocytosis of the microbes by the epithelial cells, or being transferred through specialized epithelial cells of the Peyer's patches called the microfold cells in the form of endosomes with the specific pathogen or probiotic rich population. If the luminal gut contents presented by the antigen presenting cells are rich in probiotic constituents, it results in suppression of T cell response and IgA secretion. If pathogenic constituents are presented, the T cell responses or humoral responses (via the release of specific cytokines) are displayed. The production of specific cytokines is based on the type of microbes they are exposed to (either pathogenic or probiotic). Also, the presence of beneficial probiotic organisms such as *Bifidobacteria* and *Lactobacilli* along with the supplementation of prebiotics such as inulin and oligofructose can cause a decline in formation of aberrant crypts of the colon and pH of cecum. In addition, they decrease the secretion of pro-inflammatory IL-2 and oncogenic iNOS and enhance production of SCFAs leading to decreased inflammation and reduced tumor growth. To exert such responses, the probiotics should stay in the gut for a minimum of 48 to 72 h after being supplemented [58-61] (Fig. 1). Resembling these information, a randomized double-blind placebo-controlled trial found that consuming the probiotic mixture of 30 × 10^10^ CFU *Lactobacillus* and *Bifidobacteria* for six months led to decrease in levels of cytokines such as TNF-α, IL-6, IL-10, IL-12, IL-17A, IL-17C and IL-22, whereas, there was no significant changes in IFN-γ levels in serum of CRC patients [62].

Fecal Microbiota Transplantation (FMT) is an effective alternative strategy for modulation of gut microbiota, yet in infancy at this stage. It has the ability to enhance or influence the effects of CTLA-4- and PD-1-targeting checkpoint inhibitors in a targeted immunotherapeutic approach and can effectively reduce tumor volume in mice models [63-65]. As per experimental outcomes, the effects of FMT using bacterial samples from healthy human donors along with anti-PD-1 therapy on CT26 induced female BALB/c CRC mice model were assessed. FMT increased the expressions of *Bacteroides thetaiotaomicron*, *B. fragilis*, and *Bacteroides cellulosilyticus* besides downregulating the expression of *Bacteroides ovatus*. These changes in microbiota can improve the effects of anti-PD-1 therapy by expression of metabolites such as punicic acid and aspirin [66]. Also, FMT from healthy BALB/c mice into AOM/DSS induced male BALB/c mice CRC model through enema reduced the enlargement of tumor foci and improved the survival of tumor mice. Also, it altered the dysbiotic microbiota of the intestine, recruited immune cells to TME, influenced the expression of cytokines and boosted the immune response to CRC resulting in an enhanced antitumor effect [67]. Since the response to immunotherapy has been accompanied by the abundance of bacteria such as *Akkermansia municiphila* and *Ruminococcus* spp., reinstating the sensitivity towards immunotherapy by FMT can be beneficial for CRC patients [68]. In yet another study, FMT from healthy BALB/c donor mice was done via oral administration to CT26 CRC induced BALB/c mice after the treatment with FOLFOX regimen. The severity of diarrhea and intestinal injury caused by FOLFOX regimen were reversed by FMT. In addition, upregulated expressions of the toll-like receptors (TLRs), MyD88, and serum IL-6 caused by FOLFOX regimen were alleviated by FMT [69]. At the clinical level, a randomized clinical trial involving FMT plus tislelizumab and fruquintinib as third-line or above treatment enhanced the survival of CRC patients with adaptable safety outcomes. With regard to microbiota, the enhanced abundance of *Proteobacteria* and *Lachnospiraceae* family and limited abundance of *Actinobacteriota* and *Bifidobacterium* were observed [70].

### Inhibition of CRC Cell Proliferation by Live Lactic Acid Producing Bacteria and the Cell-Killing Mechanisms

**Preclinical experiments for anti-CRC effects by exposure to *Lactobacillus* spp.**

**In vitro experiments.** *Lactobacillus plantarum* as a probiotic component was treated with curcumin and treated alone against HT-29 cells, which resulted in an IC_50_ value of 181.7 and 116.3 μl/ml after 48 h of treatment. The prebiotic like effects of curcumin influenced the metabolites of the probiotics and instigated their anticancer effects [71]. The exopolysaccharides secreted by *Lactobacillus delbrueckii* ssp. *bulgaricus* B3 and *Lactobacillus acidophilus* KLDS1.0901 possessed anti-proliferative effects against HT-29 and Caco-2 cells [72, 73]. Also, live strains of *L. acidophilus* and *Lactobacillus casei* enhanced the apoptosis-inducing behavior of 5-FU by 40% when used in combination as an adjuvant against LS513 cells [74]. Another probiotic bacteria *Lactobacillus kefiri* induced mitochondrial dysfunction, decreased the mitochondrial membrane potential (MMP) and Bcl-2 expression in AGS gastric cancer cells [75].

Suggestively, probiotic constituents have been known to pose anticancer effects on CRC cells. In this regard, *Lactobacillus fermentum* cell-free supernatant was shown to exert cytotoxic effects on 3D tumor spheroids of HCT-116 cells [76]. Also, ferrichrome, a tumor-suppressive molecule produced by *L. casei* ATCC334 strain exhibited anti-proliferative effects and induced cell death of Caco2, SKCO-1 and SW620 cells via the activation of *c-jun* N-terminal kinase (JNK) signaling pathway mediated apoptosis. In vivo, SW620 cells were injected into BALB/c nude mice where the tumor volume decreased after the administration of ferrichrome. The expressions of cleaved caspase-3 and PARP were elevated with an increase in the volume of apoptotic cells [77]. The supernatant rich in metabolites of the *L. acidophilus* ATCC 4356 probiotic showed anti-proliferative effects on Caco-2 cells in a duration- and dose- dependent manner [78]. Similarly, the *Lactobacillus rhamnosus* GG cell-free supernatant rich in bio-active metabolites induced anti-proliferative (cytostatic) effects in HT-29 and HCT-116 cells and a mitotic arrest at G2/M phase. It also showed a synergistic effect with 5-FU on the cancer cells [79]. In a similar manner, *L. acidophilus* CICC 6074, the whole peptidoglycan extract of *Lactobacillus paracasei* subsp. paracasei M5 and the supernatant of the probiotic *L. rhamnosus* YJ1 (MK453288.1) displayed cytotoxic effects on HT-29 cells in a concentration- and duration- dependent manner [80-82].

An increase in reactive oxygen species (ROS) along with a loss in MMP had led to apoptosis in the above-mentioned CRC cell lines after treatment with the probiotic constituents. The upregulation of Bax, caspases 9 and 3 besides the downregulation of Bcl-2, Bad, Bcl-xl and p21 indicated apoptosis to be the mechanism for the cell death as the release of cytochrome C from the mitochondria into the cytoplasm was evident. Also, cell cycle arrest occurred at the Sub-G1, G0/G1 phase and G2/M phase as reported in these studies. Also, in these CRC cells, the expressions of SMAC was upregulated, whereas, the expressions of survivin, cyclin D1, cyclin E, ERBB2 and p-IκBα were downregulated. Transcriptomic approach along with quantitative PCR found that a set of genes including RASL11A, CCN1, EGR1, HAVCR2, PAK1IP1, CCL20, SLC12A3 and IL32 were upregulated, whereas, the expressions of MFSD12 and IL3RA were downregulated. The varying expressions of these genes were dependent on the engagement of apoptosis which is deemed to be the predominant mechanism for anticancer effects of probiotics or their constituents on CRC cells [71, 72, 78, 80, 81].

**In vivo experiments.** Likewise, with regard to the *in vivo* studies, oligofructose–maltodextrin-enriched (as a supplement) *L. acidophilus*, *Bifidobacteria bifidum* and *Bifidobacteria infantum* (LBB) was introduced into male Sprague-Dawley rats with CRC induced by carcinogenic 1,2- dimethylhydrazine dihydrochloride (DMH). After treatment, the abundance of genera *Pseudomonas*, *Congregibacter*, *Clostridium*, *Candidactus* spp., *Phaeobacter*, *Escherichia* and *Helicobacter* were decreased, whereas, the gut abundance of *Lactobacillus* was found to be elevated. The changes in gut microbiota were found to influence the antitumor effects with limited incidence of tumor and a decreased tumor volume. The expressions of MUC2, ZO-1, occludin, and TLR2 were upregulated, whereas the expressions of TLR4, caspase 3, Cox-2, and β-catenin were decreased. This research work displayed that the modification of gut microbiota with probiotic supplementation enhanced the gut barrier and exhibited TLR2 signaling as the mechanism for CRC cell killing [83].

Similar effects were observed in in vivo tumor model (female BALB/c mice) for colon cancer as a significant volume of tumor (80%) was reduced after the administration of live *L. casei*. The upregulation of apoptosis inducing ligand TRAIL and downregulation of survivin, cyclin D1 and BIRC5a was observed which indicated the pro-apoptotic behavior of *L. casei* [84]. Similarly, *Lactobacillus rhamnosus* GG CGMCC 1.2134 (LGG) strain inhibited the proliferation of cancerous cells in DMH-induced CRC of Sprague-Dawley rats as displayed by the upregulated expressions of apoptotic genes such as Bax, caspase 3 and p53. In addition, the downregulated expressions of anti-apoptotic Bcl-2, β-catenin and the inflammation-related proteins such as NFkB-p65, COX-2 and TNFα were observed. The cell death was induced by induction of apoptosis and amelioration of inflammation. The probiotic supplementation also decreased tumor incidence, multiplicity and tumor volume [85].

Interestingly, genetically modified lactic acid bacteria (six different strains belonging to the species of *Streptococcus thermophiles* and *Lactococcus lactis*) with the ability to produce antioxidant enzymes and the anti-inflammatory cytokine IL-10 were found to exert antitumor effects in BALB/c female mice treated with DMH and reduced the occurrence of inflammation. The administration of the probiotic strain decreased damages that occurred to intestine, modified the antioxidant enzymes and produced anti-inflammatory cytokines which can decrease the extent of inflammation associated with TME of CRC in mice models [86]. Moreover, the oral inoculation of *L. acidophilus* NCFM (La) in female BALB/cByJ mice subcutaneously injected with CT-26 colon cancer cells reduced the tumor volume by 50% and decreased the severity of crypt damage. The downregulation of CXCR4 mRNA and MHC class I in addition to induction of apoptosis (increased levels of caspases 3 and 9 along with decrease in Bcl-2 levels) in tumor tissue was observed [87] (Table 2).

**Preclinical experiments for anti-CRC effects by exposure to *Bifidobacterium* spp.**

A cocktail of 5 strains of *Bifidobacteria* were shown to cause a decline in expressions of genes involved in tumor progression such as EGFR by 4.4 folds, HER-2 by 6.7 folds, and PTGS-2 by 20 folds and induced apoptosis in LS174T CRC cells. The cocktail decreased the average disease activity index in AOM/DSS induced female BALB/c mice CRC model in addition to reducing incidence of tumor, the tumor volume and increasing the colon length. The mice treated with the cocktail had limited inflammation of the colon and the possessed a lower grade tumor in comparison to untreated tumor mice [88]. Also, probiotic supplementation with *Bifidobacterium animalis* subsp. *lactis* SF can enhance the antitumor effects of the antitumor drug irinotecan by modifying the gut microbiota (reducing the incidence of pro-inflammatory microbiota) and decrease intestinal inflammation. There was a reduction in seepage of TGF-β and inhibition of PI3K/Akt pathway. CPT-11 mediated immunosuppression was curbed and CD4^+^ and CD8^+^ T cells linked pro-inflammatory environment was established in the TME disturbing the tumor proliferation and invasive ability [89].

Cell-free supernatants of five different *Bifidobacteria* strains were shown to exert cytotoxic effects on HT-29 and Caco-2 cells (IC_50_ range of 65 μg/ml to 80 μg/ml). *B. bifidum* showed the highest percentage of early and late apoptosis in both HT-29 and Caco-2 cells. The expression of pro-apoptotic BAD, caspases-3, -8, -9, and Fas-R genes were upregulated after treatment with the supernatant besides the downregulation of Bcl-2 gene [90]. Similar effects of metabolites from cell-free supernatant of *Bifidobacterium* (99% similar to *B. bifidum* strain BXH2-3) were observed against SW742 CRC cells. The cancer cells were fragmented, the cellular density was reduced and cell death happened by toxin cytopathic activities as they floated on the surface of the medium. DNA fragmentation was observed as a measure of apoptosis [91]. In addition, the metabolite produced by *Bifidobacterium pseudolongum* termed inosine can augment the antitumor effects of checkpoint blockade immunotherapy (intra-tumoral CD8^+^ T-cell activation) through the stimulation of human adenosine receptor (A_2A_) of the T cells in Azoxymethane (AOM)/Dextran sodium sulfate (DSS) induced intestinal tumors of SPF mice [92]. Also, the colon bacteria *Bifidobacterium adolescentis* SPM0212 inhibited the proliferation of HT-29, SW 480, and Caco-2 CRC cells in a dose-dependent manner. The microbe enhanced the TNF-β production and caused changes in cellular morphology of RAW 264.7 macrophages [93].

The mechanism of antitumor activity of *Bifidobacteria* is by converting polyunsaturated fatty acids into prebiotic like pectic oligosaccharides which may delay tumor progression and cause an increase in another bacterial genus such as *Bacteroides*. This process termed as biotransformation can also improve the therapeutic efficacy of antitumor drugs or plant-based drinks by converting them into active antitumor molecules. The bacterial species can also reduce the levels of carcinogenic molecules (thereby preventing carcinogenesis) and inhibit tumor growth and aid in tumor regression. They can also inhibit the growth of coliforms and thereby improve the nutrient availability for probiotics by reducing the competition for nutrients by such pathogenic coliforms as they can attach to cells of the intestine effectively. This can provide more space and nutrients for useful microbes to grow. In addition, they can generate free radicals to kill pathogenic microbes, display anti-mutagenic activities and increase the secretion of anti-inflammatory cytokines [94] (Table 3).

### Anti-CRC Activity of Microbial Metabolites or Postbiotics

**Anti-CRC effects of conjugated linoleic acid**

Probiotic organisms can convert linoleic acid (sourced from vegetable oils, nuts, seeds, meats, and eggs) into conjugated linoleic acids (CLA), which has been shown to possess anticarcinogenic and antitumor activities *in vivo* [95, 96]. Supporting this statement, CLA inhibited the proliferation of HT-29 cells, repressed the synthesis of genetic material and induced apoptosis of the CRC cells. It also decreased the expressions of ErbB2 and ErbB3 at genome and proteome levels and downregulated the activity of PI3-kinase/Akt pathway [97]. In yet another study, the CLA isomer t10c12 also caused a decline in DNA synthesis and induced apoptosis in Caco-2 cells. The production of IGFBP-2, can induce proliferation of tumors, was decreased along with the decrease in insulin growth factor (IGF-II) secretion [98]. Also, *cis*-9 (9Z), *trans*-11 (11E)-CLA showed inhibitory effects on proliferation of HT-29 and Caco-2 cells and caused a decline in expressions of *c-myc*, cyclin D1, *c-jun* and PPAR-δ in a dose-dependent manner. Therefore, the inhibitory effects of the CLA isomers are due to the downregulation of expressions of constituents of APC-β-catenin-TCF-4- and PPAR-δ signaling [99].

Probiotic mixture VSL3 consists of four strains of *Lactobacillus* such as *Lactobacillus acidophilus*, *Lactobacillus plantarum*, *Lactobacillus casei*, and *Lactobacillus delbrueckii* *subspecies bulgaricus*, three strains of *Bifidobacterium* such as *Bifidobacterium breve*, *B. longum*, and *Bifidobacterium infantis* and one strain of *Streptococcus salivarius* subspecies *thermophilus* [100]. This mixture was known to produce CLA from linoleic acid (100-fold higher production in comparison to mice group without the supplementation of the probiotic mixture in 129/SvEv mice) which caused a decline in the viability of HT-29 and Caco-2 cells and induced apoptosis in the CRC cells. The expressions of PPARγ were elevated which can result in the decrease of secretion of pro-inflammatory factors. Therefore, CLA can attenuate inflammation and prevent CRC [101].

Also, CLA from a probiotic *Pediococcus pentosaceus* GS4 enhanced the expression of PPARγ in HCT-116 CRC cells. The expressions of Prostaglandin E2, COX-2 and 5-LOX were reduced and the cell death was caused by mitochondrial depolarization, intrinsic apoptosis in a PPARγ-dependent manner with cell cycle arrest happening at the G0/G1 stage [102]. Yet another research on the same probiotic strain by the same research group indicated that CLA induced cell death of HCT-116 cells by apoptosis and evading the involvement of NF-κB and p-Akt. In AOM-induced CRC mice model, the severity of the disease was reduced, along with a decrease in oxidative stress and oncogenic properties. The induction of apoptosis in colon cells was mediated by PARP cleavage, increase in caspase 3 levels, DNA fragmentation and inhibition of the activity of histone deacetylases [103]. In a pilot level clinical study, oral administration of CLA in the form of capsules decreased the serum TNF-α, hsCRP, and MMP-9 levels and maintained the levels of interleukin-6 at bay in rectal cancer patients. The exposure to polyunsaturated fatty acid can reduce tumor invasion and resistance to therapy in rectal cancer patients [104]. As a concluding remark, CLA and its isomers possess antitumor, anti-mutagenic and anti-oxidant properties [105] (Table 4).

**Anti-CRC effects of the SCFA butyrate**

The SCFA butyrate can maintain the function of intestinal epithelial barrier by causing a trigger in MUC2 levels of LS174T CRC cells and reduce the intestinal transport levels causing a decline in CRC progression. Butyrate can also induce apoptosis in CRC cells mediated by the involvement of Wnt signaling. It can affect the invasive and colony-forming abilities of CRC cells by upregulating the expressions of miR-203, bax, P21waf1 and endocan and accumulation of β-catenin. Also, it can enhance the expression of p57 and suppress the formation of c-Myc causing a decrease in levels of oncogenic miR-17-92a. Besides, it can also decrease the expression of histone deacetylase 3 (HDAC3) causing a decline in tumor progression [106]. Also, *L. plantarum* strain S2T10D exerted anti-proliferative effects and repression of metabolic activities of HT-29 cells owing to their butyrogenic ability via the downregulation of cyclin B1 and D1 along with inducing cell cycle arrest at G2/M phase [107]. Moreover, butyrate can positively control the antitumor CD8^+^ T cell response in mice model and increase the effectiveness of chemotherapy in an ID2-dependent mode of IL-12 signaling [108].

Clinically, patients who respond well to immunotherapy and chemotherapy, showed a relatively higher abundance of butyrate-producing microbiota and showed increased fecal and plasma levels of SCFAs. SCFAs such as butyrate can reduce the intratumoral levels of T cells, INF-γ and TNF-α and cause the tumor cells to proliferate at a slower rate with better immune response. The levels of butyrate-producing microbes consistent with higher fecal levels of SCFAs can result in better response to radiotherapy and improve radiosensitivity of the tumor [109]. Butyrate is in shorts the most active and potent histone deacetylase inhibitor among compounds derived from natural sources. It can cause a decline in activities of NF-κB and STAT3 signaling and downregulate the expressions of bcl-2, bcl-XL, *c-myc*, cyclin D1, and HIF-1. This can lead to decreased cellular proliferation and induce apoptosis under hypoxic environments such as TME [110]. Likewise, organoids generated from resected human CRC tumors were treated with 50 mM butyrate and 50 mM propionate which increased the population of CD8^+^ T cells in the TME [111].

In azoxymethane (AOM)-induced male Sprague–Dawley CRC rat model, the tumor growth was lower after being orally fed with butyrylated 10% high-amylose maize starch. The tumor incidence and the number of tumors were significantly less in the prebiotic-treated group where the plasma butyrate levels were higher [112]. *Lactobacillus reuteri* derived supernatant rich in SCFAs reduced the abundance of pathogenic *Proteobacteria* and *Fusobacterium* and increased the abundance of useful *Bacteroidetes* and *Firmicutes* in CT26-bearing male Balb/C mice CRC model. The increased expressions of pro-apoptotic caspase-3 and Bax and the downregulation of anti-apoptotic Bcl-2 was observed in CT26 tumor cells treated with enriched occurrence of butyrate producing microbiota which can effectively bind to G protein-coupled receptor 109A (GPR109A). The enhanced accumulation of butyrate and GPR109A led to enhanced levels of binding and increased apoptotic levels *in vivo* [113]. Similarly, in AOM/DSS-induced male BALB/c mice CRC model, butyrate administration improved the loss in weight, enlarged the colon length, limited the thickening of the walls of intestine and reduced intestinal inflammation. It reversed the gut microbial composition and protecting the loss of *Actinobacteriota* with antitumor and antibacterial activities and improved the abundance of genera *Bacteroidia* and *Clostridia* along with the abundance of *Bifidobacteriaceae* at the family level. Butyrate changed the dysbiotic microbial environment of the CRC model’s gut to a healthier one [114].

The combination of SCFAs such as 67.5 mM acetate, 40 mM butyrate, 25.9 mM propionate administered orally into colitis-associated AOM/DSS-induced CRC male BALB/c mice model decreased the tumor incidence and size, improved inflammation of the colon and suppressed the expression of proinflammatory cytokines such as IL-6, TNF-α and IL-17 [115]. The same combination at different test concentrations taking into account the IC_50_ such as acetate (81.04 mM), butyrate (10.84 mM), propionate (32.25 mM) for RKO cells and 89.52 mM, 4.57 mM and 22.70 mM, respectively for HCT-15 inhibited the growth of such CRC cells at the tested dose. The SCFAs inhibited the colony forming abilities and proliferation of the cells in addition to induction of apoptosis in both the cell lines. The cell death in RKO cells was more due to the effects of acetate, whereas the effects in HCT-15 cells were associated to the effects of butyrate. The apoptotic cell death was associated more to lysosomal-membrane permeabilization and acidification of the cytosolic components of the cancerous cells [116]. Similarly, *E. coli* KUB-36, an isolate of healthy human gut, produced SCFAs including acetic acid (23.89 mM), caproic acid (3.63 mM), butyric acid (3.01 mM), valeric acid (2.83 mM), iso-valeric acid (2.78 mM), propionic acid (2.15 mM) and iso-butyric acid (1.69 mM). The cytotoxic effects of the metabolites displayed as an outcome of IC_50_ was 58.19%, 55.31% and 50.68% after 24, 48 and 72 h of treatment. Acetic acid and butyric acid were effective in cytotoxic effects on HT-29 cells in comparison to other metabolites. The *E. coli* KUB-36 metabolites such as acetic acid, butyric acid, valeric acid and isovaleric acid resulted in the suppression of inflammatory cytokines IL-1β, IL-6, IL-8 and TNF-α expressions [117].

**Fig. 2:**
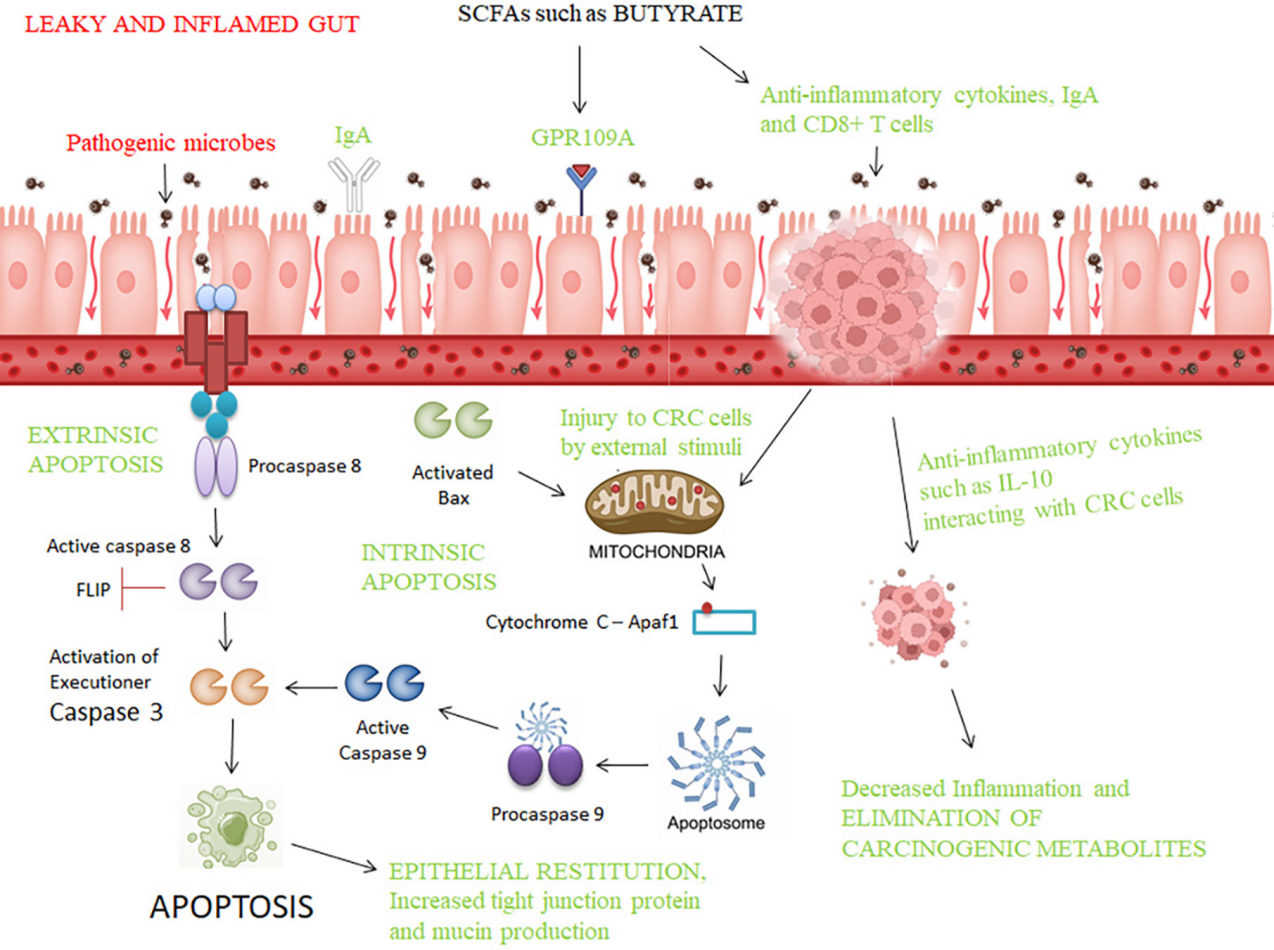
Interactions of SCFAs with CRC cells and the cell-killing mechanisms.

Also, a combination of acetate, propionate, and butyrate was treated against the Caco2 cells. Propionate and butyrate were more cytotoxic than acetate and the SCFAs elevated the ROS levels in the CRC cells. The expression of metabolites showed the levels of leucine, glycine, phenylalanine, tyrosine, choline, fructose, acetylcholine to be increased and the levels of ATP/ADP, lactate, choline, UDP glucuronate and UDP glucose to be decreased. The cells treated with propionate and butyrate showed increases in levels of metabolites such as acetate, glucose, α-keto-β-methyl-valerate (α-KMV), isoleucine, succinate and nicotinamide adenine dinucleotide (NAD+), whereas, the levels of glutathione, phosphocholine and taurine were reduced. With regard to the transcriptome signatures, the pathways associated with organic acid transport and catabolism, ROS metabolism, amino acid transport, and glutamine family amino acid catabolism were upregulated, whereas, the pathways linked to mitochondrial gene expression, oxidative phosphorylation, and amino acid activation and methylation were downregulated [118]. The anticancer effects of the SCFA butyrate are presented in Table 5. The anticancer effects and the mechanisms of such effects for probiotics and postbiotics are represented in Fig. 2.

## Conclusion

The microbiome of the GI tract remains a key player in maintaining the health and disease status of a host. The useful microbes can compete with the dysbiotic microbes for their residing the host gut. Microbial markers at genomic, proteomic and metabolic levels have been identified every day for early diagnosis of colorectal cancer. Probiotics such as lactic acid bacteria, *Bifidobacteria*, *Firmicutes* and *Saccharomyces* can help maintain eubiosis when administered in appropriate amounts as a part of diet and this process determines the composition of gut microbiome. Also, the type of food intake, sex, age, concomitant comorbid conditions, treatment undertaken, intestinal environment (pH, oxygen levels and availability of nutrients) and the environment of the host decide the type of microbial population of the gut. Furthermore, probiotics have been shown to pose antitumor effects in both in vitro and *in vivo* models through the induction of apoptosis and other key mechanisms, and alleviate cancer-related symptoms in humans. To conclude, probiotics are nontoxic to use in humans even consumed at higher volumes and are to be studied further in promoting their appliance at clinical levels.

## Figures and Tables

**Table 1:** Microbial community rich in various sample sources of CRC patients according to metagenomic analysis.

Sample source	Microbial community	Reference
Gut and feces	*Actinomyces*, *Bacteroides fragilis*, *Bifidobacterium*, *Clostridium hylemonae*, *Clostridium symbiosum*, *Enterococcus faecalis*, *Escherichia coli*, *Fusobacterium nucleatum*, *Gemella morbillorum*, *Lachnoclostridium*, *Parvimonas micra*, *Peptostreptococcus stomatis*, *Porphyromonas asaccharolytica*, *Pseudomonas*, *Roseburia*, *Ruminococcus*, *Salmonella*, *Solobacterium moorei* and *Streptococcus bovis*	[20]
Intra-tumoral region, oral cavity and feces	*Fusobacterium nucleatum*, *Escherichia coli*, *Clostridium symbiosum*, *Bacteroides fragilis*, *Actinomyces*, *Streptococcus*, *Peptostreptococcus*, *Porphyromonas* and *Parvimonas micra*	[21]
Feces and oral cavity	*Fusobacterium*, *Enterococcus*, *Porphyromonas*, *Salmonella*, *Pseudomonas*, *Peptostreptococcus*, *Actinomyces*, *Bifidobacterium*, *Roseburia*, *Treponema denticola* and *Prevotella intermedia*	[22]
Feces	*Lachnospiraceae*, *Ruminococcaceae*, *Erysipelotrichaceae*, *Peptostreptococcaceae*, *Christensenellaceae*, *Defluviitaleaceae*, *Clostridiaceae*, *Streptococcaceae*, *Veillonellaceae*, *Bacteroidaceae*, *Rikenellaceae*, *Porphyromonadaceae*, *Pasteurellaceae*, *Enterobacteriaceae*, *Synergistaceae*, *Bifidobacteriaceae*, *Fusobacteriaceae*	[23]
Saliva, feces and cancer tissues	*Bacteroides*, *Roseburia*, *Ruminococcus*, *Oscillibacter*, *Alistipes*, *Akkermansia*, *Halomonas*, *Shewanella*, *Faecalibacterium*, *Blautia*, *Clostridium*, *Firmicutes*, *Bacteroidetes*, *Proteobacteria*, *Fusobacteria*, *Actinobacteria*, *Parvimonas*, *Peptostreptococcus*, *Alistipes*, and *Escherichia*, *Streptococcus*, *Helicobacter pylori*, *Enterococcus faecalis* and *Bacteroides fragilis*	[24]

**Table 2:** Anticancer effects of the probiotic *Lactobacillus* and its constituents.

Organism	Cancer cell/model	Genes involved	Mechanism of activity	Reference
In vitro experiments				
*Lactobacillus plantarum*	HT-29 cells	Upregulation of Bax, caspases 9 and 3 besides the downregulation of Bcl-2	Apoptosis and cell cycle arrest at Sub-G1 stage	[71]
Exopolysaccharides secreted by *Lactobacillus delbrueckii*	HT-29 cells	Increased expressions of pro-apoptotic genes Bax, Caspases 3 and 9 and decreased expressions of anti-apoptotic genes Bcl- 2 and Survivin	Apoptosis	[72]
*Lactobacillus acidophilus*	HT-29 cells	loss in MMP, upregulation of genes such as RASL11A, CCN1, EGR1, HAVCR2, PAK1IP1, CCL20, SLC12A3, IL32, and downregulated expressions of MFSD12 and IL3RA	Apoptosis	[73]
*Lactobacillus acidophilus* and *Lactobacillus casei*	LS513 cells	Upregulation of caspase 3 and downregulation of p21 protein	Apoptosis	[74]
*Lactobacillus kefiri*	AGS cells	Induced mitochondrial dysfunction, decreased mitochondrial membrane potential (MMP) and Bcl-2 expression	Apoptosis	[75]
*Lactobacillus fermentum* cell-free supernatant	3D tumor spheroids of HCT-116 cells	1. upsurge in the volume of apoptotic cells 2. Upregulated expressions of pro-apoptotic Bax and cleaved caspase 3 and the downregulated expressions of antiapoptotic Bcl-2 and p-IκBα	Apoptosis	[76]
Ferrichrome, a tumor-suppressive molecule produced by *Lactobacillus casei* strain	Caco2, SKCO-1 and SW620 cells, SW620 cells injected into BALB/c nude mice	1. Decrease in tumor volume 2. Elevated expressions of cleaved caspase-3 and PARP 3. Increase in the volume of apoptotic cells	JNK signaling pathway mediated apoptosis	[77]
Supernatant rich in metabolites of *Lactobacillus acidophilus*	Caco-2 cells	Upregulated expression of SMAC and downregulation of survivin	Apoptosis	[78]
*Lactobacillus rhamnosus* GG cell-free supernatant	HT-29 and HCT-116 cells	cytostatic effects	Mitotic arrest at G2/M phase	[79]
Whole peptidoglycan extract of *Lactobacillus paracasei*	HT-29 cells	1. Upregulated expressions of pro-apoptotic genes such as Bax and Bad in addition to downregulated expressions of anti-apoptotic Bcl-xl 2. Release of cytochrome C from the mitochondria into the cytoplasm	Apoptosis	[80]
Supernatant of *Lactobacillus rhamnosus*	HT-29 cells	1. Upregulated expressions of pro-apoptotic Bax, caspases 3 and 9 besides a decrease in expression of anti-apoptotic Bcl-2 2. Decrease in expressions of cyclin D1, cyclin E, and ERBB2 2. Growth arrest at G0/G1 phase	Apoptosis	[81]
*Lactobacillus acidophilus*	HT-29 cells, tumorbearing female BALB/c mice	1. loss in MMP and release of cytochrome C 2. upregulated expressions of pro-apoptotic Bax, caspases 3 and 9 and downregulated expression of anti-apoptotic Bcl-2	Apoptosis	[82]
In vivo experiments				
*Lactobacillus acidophilus*, *Bifidobacteria bifidum* and *Bifidobacteria infantum*	CRC-bearing male Sprague-Dawley rats	1. The abundance of genera *Pseudomonas*, *Congregibacter*, *Clostridium*, *Candidactus* spp., *Phaeobacter*, *Escherichia* and *Helicobacter* were decreased, whereas, the gut abundance of *Lactobacillus* was found to be elevated 2. The expressions of MUC2, ZO-1, occludin, and TLR2 were upregulated, whereas, the expressions of TLR4, caspase 3, Cox-2, and β-catenin were decreased	TLR2 signaling	[83]
*Lactobacillus casei*	CRC-bearing female BALB/c mice	Upregulation of TRAIL and downregulation of survivin, cyclin D1 and BIRC5a	Apoptosis	[84]
*Lactobacillus rhamnosus*	CRC-bearing Sprague-Dawley rats	1. Upregulated expressions of Bax, caspase 3 and p53 2. Downregulated expressions of Bcl-2, β-catenin and the inflammation-related proteins such as NFkB-p65, COX-2 and TNFα	Apoptosis	[85]
Six different strains belonging to the species of *Streptococcus thermophiles* and *Lactococcus lactis*	CRC-bearing female BALB/c mice	Production of antioxidant enzymes and the antiinflammatory cytokine IL-10	Anti-inflammatory effects	[86]
*Lactobacillus acidophilus*	CRC-bearing female BALB/cByJ mice	1. Decrease in the tumor volume by 50% and the severity of crypt damage 2. Downregulation of CXCR4 mRNA and MHC class I molecules 3. Increased levels of caspases 3 and 9 and decrease in Bcl-2 levels	Apoptosis	[87]

**Table 3:** Anticancer effects of the probiotic *Bifidobacterium* and its constituents.

Organism	Cancer cell/model	Cellular effects	Mechanism of activity	Reference
Probiotic organisms				
A cocktail of 5 strains of *Bifidobacteria*	LS174T cells, AOM/DSS induced female BALB/c mice model	1. Decline in expressions of genes involved in tumor progression such as EGFR, HER-2 and PTGS-2 2. reduced incidence of tumor, the tumor volume and increase in the colon length	Apoptosis and reduced inflammation of the colon	[88]
*Bifidobacterium animalis* subsp. *lactis* SF	HCT-8 cells	1. Reduction in seepage of TGF-β and CPT-11 mediated immunosuppression 2. Increase in the volume of CD4^+^ and CD8^+^ T cells	Promotion of apoptotic autophagy and Inhibition of PI3K/AKT pathway	[89]
Probiotic constituents				
Cell-free supernatants of *Bifidobacteria bifidum*	HT-29 and Caco-2 cells	Upregulated expressions of proapoptotic BAD, caspase-3, caspase- 8, caspase-9, Fas-R genes and downregulated expressions of Bcl-2	Apoptosis	[90]
Cell-free supernatant of *Bifidobacteria bifidum*	SW742 cells	Decrease in cellular density and DNA fragmentation	Apoptosis	[91]

**Table 4:** The anticancer effects of the postbiotic conjugated linoleic acid.

Cancer cell/model	Molecular effects	Reference
HT-29 cells	1. decreased expressions of ErbB2 and ErbB3 besides the downregulated activity of PI3-kinase/Akt pathway 2. Repression of DNA synthesis and induction of apoptosis	[97]
Caco-2 cells	1. Decline in DNA synthesis and induction of apoptosis 2. Decrease in the production of IGFBP-2 and IGF-II	[98]
HT-29 and Caco-2 cells	Declined expressions of *c-myc*, cyclin D1, *c-jun* and PPAR-δ (constituents of APC-β-catenin-TCF-4- and PPAR-δ signaling)	[99]
HT-29 and Caco-2 cells	Increased expressions of PPARγ and induction of apoptosis	[101]
HCT-116 cells	1. Upregulated expression of PPARγ and downregulated expressions of Prostaglandin E2, COX-2 and 5-LOX 2. Induction of apoptosis and cell cycle arrest at the G0/G1 stage	[102]
HCT-116 cells, AOM-induced CRC mice model	1. Induction of apoptosis avoiding the involvement of NF-κB and p-Akt 2. PARP cleavage, increase in caspase 3 levels, DNA fragmentation and inhibition of the activity of histone deacetylases	[103]
Clinical patients with rectal cancer	Decrease in the serum levels of TNF-α, hsCRP, and MMP-9 levels and maintenance of IL-6 levels	[104]

**Table 5:** The postbiotic SCFA butyrate and its anticancer effects.

Cancer cell/model	Molecular effects	Reference
LS174T cells	1. Trigger in MUC2 levels 2. Induction of apoptosis mediated by the involvement of Wnt signaling 3. Upregulated expressions of miR-203, bax, P21waf1 and endocan along with the accumulation of β-catenin 4. Increase in expression of p57, decrease in formation of c-Myc and the levels of HDAC3 and oncogenic miR-17-92a	[106]
HT-29 cells	1. Downregulation of cyclin B1 and D1 2. Induction of cell cycle arrest at G2/M phase	[107]
Mice models including C57BL/6J	1. Increase in CD8^+^ T cell response 2. Increase in effectiveness of chemotherapy via the involvement of ID2-dependent mode of IL-12 signaling	[108]
Clinical patients of CRC	1. reduction in the intratumoral levels of T cells, INF-γ and TNF-α 2. Better immune response and improved response to radiotherapy and improvement in radiosensitivity	[109]
Clinical patients of CRC	1. Decline in activities of NF-κB and STAT3 signaling and induction of apoptosis 2. Downregulation of the expressions of bcl-2, bcl-XL, c-myc, cyclin D1 and HIF-1	[110]
Organoids generated from resected human CRC tumors	Increase in the population of CD8^+^ T cells	[111]
(AOM)-induced male Sprague–Dawley CRC rat model	Decrease in tumor incidence and the number of tumors	[112]
CT26-bearing male Balb/C mice CRC model	1. Decrease in the abundance of pathogenic *Proteobacteria* and *Fusobacterium* and increase in the abundance of useful *Bacteroidetes* and *Firmicutes* 2. Increased expressions of pro-apoptotic caspase-3 and Bax and the downregulation of anti-apoptotic Bcl-2 resulting in the induction of apoptosis	[113]
AOM/DSS-induced male BALB/c mice CRC model	1. Improved the loss in weight, enlarged the colon length, limited the thickening of the walls of intestine and reduced intestinal inflammation 2. improved abundance of the genera *Bacteroidia* and *Clostridia*	[114]
AOM/DSS-induced CRC male BALB/c mice model	1. decrease in the tumor incidence and size, decrease in inflammation of the colon 2. Decline in the expressions of proinflammatory cytokines such as IL-6, TNF-α and IL-17	[115]
RKO cells and HCT-15 cells	1. Inhibition of the colony forming abilities 2. The induction of apoptosis	[116]
HT-29 cells	suppression of inflammatory cytokines IL-1β, IL-6, IL-8 and TNF-α expressions	[117]
Caco2 cells	1. The expressions of metabolites such as leucine, glycine, phenylalanine, tyrosine, choline, fructose and acetylcholine were increased, whereas, the levels of ATP/ ADP, lactate, choline, UDP glucuronate and UDP glucose were decreased 2. Increases in levels of metabolites such as acetate, glucose, α-keto-β-methylvalerate (α-KMV), isoleucine, succinate and nicotinamide adenine dinucleotide (NAD+), in addition to the decrease in levels of glutathione, phosphocholine and taurine	[118]
